# Estrogen Receptor Alpha–Expressing Neurons in Bed Nucleus of the Stria Terminalis and Hypothalamus Encoding Aggression and Mating

**DOI:** 10.1523/ENEURO.0218-24.2024

**Published:** 2024-11-26

**Authors:** Wen-Qiu Wang, He-Xin Zhao, Xiao-Lin Shen, Li-Zhang Zeng, Hong-Yan Geng

**Affiliations:** ^1^Key Laboratory of Brain, Cognition and Education Science, Ministry of Education, South China Normal University, Guangzhou 510631, China; ^2^Institute for Brain Research and Rehabilitation, and Guangdong Key Laboratory of Mental Health and Cognitive Science, South China Normal University, Guangzhou 510631, China

**Keywords:** aggression, estrogen receptor alpha–expressing neurons, mating, medial preoptic area, the principal nucleus of the bed nucleus of stria terminalis, ventrolateral subdivision of the ventromedial hypothalamus

## Abstract

Aggression and mating of male mice are strongly associated with Esr1-expressing neurons in the bed nucleus of the stria terminalis (BNSTpr) and hypothalamus in the vomeronasal pathway. By projecting to the downstream hypothalamus, the upstream BNSTpr^Esr1^ gates mating and aggression of male mice and maternal behavior of female mice. The medial preoptic area (MPOA) and ventrolateral subdivision of the ventromedial hypothalamus (VMHvl) are two subdivisions of the hypothalamus downstream. In addition to receiving projections from upstream BNSTpr, there is also a mutual projection between MPOA and VMHvl. In the process of transforming sex information into mating and aggression, Esr1-expressing neurons in BNSTpr, MPOA, and VMHvl act as messengers of information, finally producing inhibitory or excitatory projection. These projections are different in direction, but they all work together to control the behavior selection that is most conducive to defense and reproduction when male mice encounter female or male mice. Here, we summarized the property and the function of connections between these Esr1-expressing neurons in BNSTpr, MPOA, and VMHvl that encode mating and aggression and highlight the importance and benefits of inhibitory projection of Esr1-expressing cells in mating and aggression.

## Significance Statement

Neural networks that control mating and aggression have been studied with particular attention to the activity of Esr1-expressing cells in recent years. It is highly expressed in regions that regulate social behavior and is critical for aggression and mating behavior in male mice. More and more studies have been conducted on the function of Esr1-expressing cells, and further studies on the subtypes of Esr1-expressing cells have gradually clarified the molecular mechanism regulating mating and aggression. Here, the opinion deepens our understanding of the function and significance of Esr1-expressing cells and underscores the need for continued research on them.

## Introduction

Social behavior can be divided into two stages: the appetitive stage and the consummatory stage. For defense and reproduction, social behavior typically transitions from the appetitive stage to the consummatory stage (Hashikawa et al., 2016). While mating and aggression take place during the consummatory stage, sniffing is a behavior that happens during the appetitive stage (B. Yang et al., 2022). In order to ensure that animals can respond appropriately to their living environment, external pheromones and internal sex steroid hormones generated by the gonads strictly regulate the neural circuits of mating and aggression (C. F. Yang and Shah, 2014).

On the one hand, sex steroid hormones, including testosterone, estrogen, and progesterone, induce selective expression of genes in neurons that express corresponding receptors including androgen receptor (AR), estrogen receptor alpha (Esr1), estrogen receptor beta (Esr2), and progesterone receptor (PR). Thus, the connection, activity, and state of the neural network may all be impacted by this dynamic regulation (Gegenhuber et al., 2022; Yu et al., 2023). Testosterone attaches to AR and can be aromatized to become estrogen, which then binds to Esr1 and Esr2. Without an efficient aromatase, male mice cannot engage in mating and aggression (Matsumoto et al., 2003). However, testosterone is more important to increase the intensity of male behavior (Juntti et al., 2010). Esr1 mediates the main effect of estrogen on behavior, whereas Esr2 has a more regulatory function (C. F. Yang and Shah, 2014). Additionally, progesterone influences female mating through PR. Both PR and Esr1 are required for female sexual receptivity (Blaustein, 2008).

On the other hand, chemical cues with external pheromones have two forms, i.e., volatile and nonvolatile; the latter is processed by the vomeronasal organ (VNO) pathway (Bergan et al., 2014; Xiao et al., 2022). Sex cues in urine are transmitted by VNO to the accessory olfactory bulb (AOB; Wagner et al., 2006; Bergan et al., 2014; Leroy et al., 2018; Li and Dulac, 2018), which in turn is further transmitted to the medial amygdala (MeA), the principal nucleus of the bed nucleus of stria terminalis (BNSTpr), and two subdivisions of hypothalamus, medial preoptic area (MPOA) and ventrolateral subdivision of the ventromedial hypothalamus (VMHvl; Dong et al., 2001; Bergan et al., 2014; B. Yang et al., 2022; Itakura et al., 2022). Years of study have improved our understanding of social behavior neural network. Attacking and sexual behavior of male mice was disrupted by damage to the VNO, AOB, MeA, BNST, or suppression-specific neurons in these nuclei, and activation of these neurons can lead to mating and aggression (Ropartz, 1968; Rowe and Edwards, 1972; Dominguez et al., 2001; Lin et al., 2011; Hong et al., 2014; Unger et al., 2015; Leroy et al., 2018; B. Yang et al., 2022).

**Figure 1.**
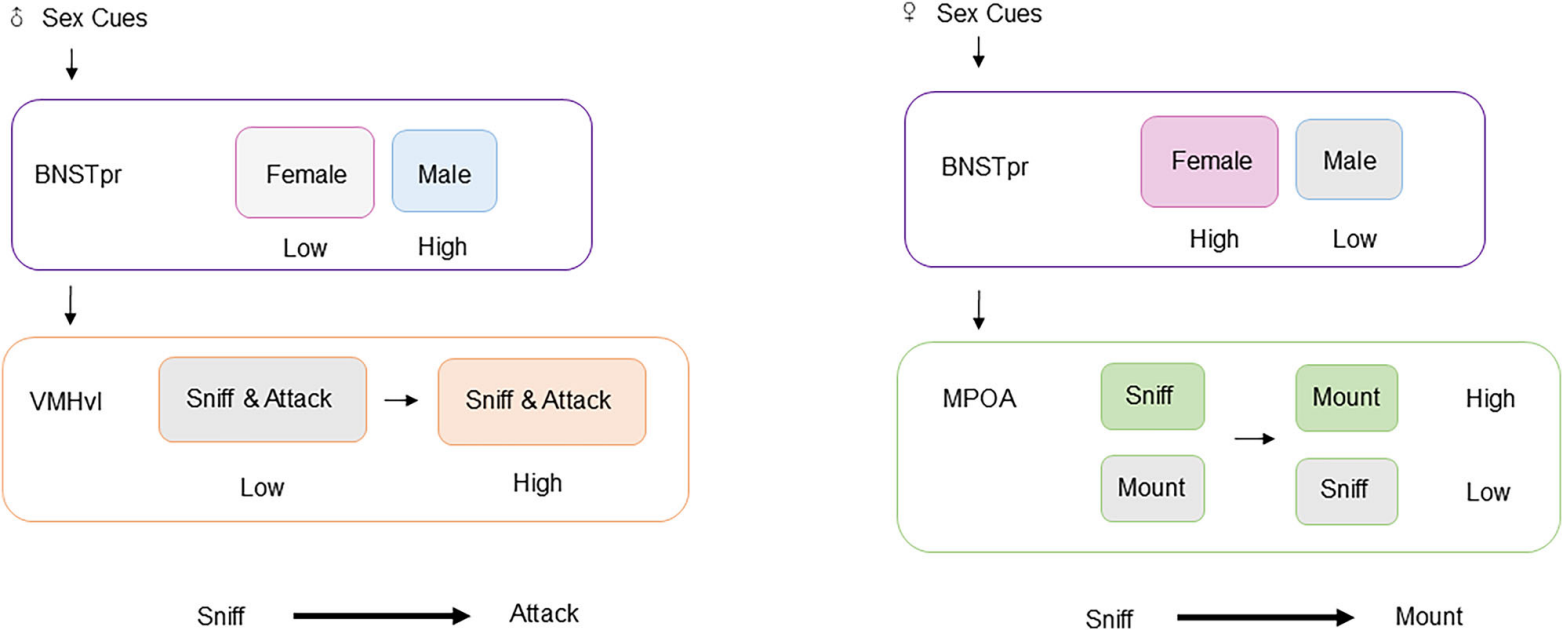
Sex cues are translated into social behavior. Male-preferring neurons in BNSTpr transmit male sex cues to VMHvl and realize the behavior transition from sniffing to aggression. Projection from BNSTpr triggers sniff- and attack-selective neurons overlapped in VMHvl during this phase. In order to realize the behavior shift from sniffing to mounting, female-preferring neurons in BNSTpr send female sex cues to MPOA. In this phase, mount-selective neurons, which are distributed independently of sniff-selective neurons, are activated while the projection from BNSTpr inhibits the sniff-selective neurons in MPOA.

In order to better understand how sex cues are transformed from convergence to divergence, we focused on the neural circuits from BNSTpr to MPOA and VMHvl in the vomeronasal pathway. Recent research has focused on the manifestation of neurons expressing estrogen receptor alpha (Esr1) in response to sex cues and behavior selection (Fig. 1). This has provided insights into the neuronal representation in response to sex and behavior selection in BNSTpr and hypothalamus, as well as the transformation of both regions and the connections between heterogeneous neuron populations in hypothalamus.

Sex hormone receptor-expressing neurons are involved in sex-dependent social behaviors to a large extent (Wu et al., 2009; C. F. Yang and Shah, 2014; B. Yang et al., 2022). In male mice, the distribution and function of Esr1- and aromatase-expressing cells significantly overlap, but the distribution of Esr1-expressing cells is larger than aromatase-expressing cells (Stanić et al., 2014). In BNST, Esr1 is primarily expressed in BNSTpr (BNSTpr^Esr1^; Mei et al., 2023). In the study of the sex-specific neural representation of BNSTpr in male mice, the researchers suspended mice of different sexes in male mice cages. During this time, the male mice in cage are not allowed to mount or attack, only to sniff. At this time, the neurons that were only activated while sniffing female or male mice were classified as female-preferring neurons and male-preferring neurons, respectively (B. Yang et al., 2022). They used single-cell resolution imaging BNSTpr^Esr1^ during mating and aggression of male mice to distinguish between male- and female-preferring neurons in BNSTpr and discovered that the ratio of female-preferring to male-preferring neurons in BNSTpr is 2:1 (B. Yang et al., 2022). Furthermore, the sex selection in BNSTpr of male mice was shown to have a greater difference compared with behavior selection. This suggests that BNSTpr plays a significant role in proceeding the sex cues rather than behavior selection, which is supported by the sex-specific responses in two overlapping subsets of BNSTpr^Esr1^, i.e., BNSTpr^Tac1^ (Tac1, tachykinin 1, a subset of BNSTpr^Aro^ neurons) and BNSTpr^Aro^ (aromatase-expressing BNSTpr^Esr1^), although BNSTpr^Esr1^ alone does not represent sex in the same way as these subsets do (Bayless et al., 2019, 2023; B. Yang et al., 2022). When the activity of upstream BNSTpr^Esr1^ is inhibited, the sex bias in downstream VMHvl is completely reversed, further highlighting the importance of BNSTpr^Esr1^ for sex recognition in male mice. It is the sexually dimorphic processing of social cues that enables BNSTpr^Esr1^ to encode mating and aggression in male mice.

**Figure 2.**
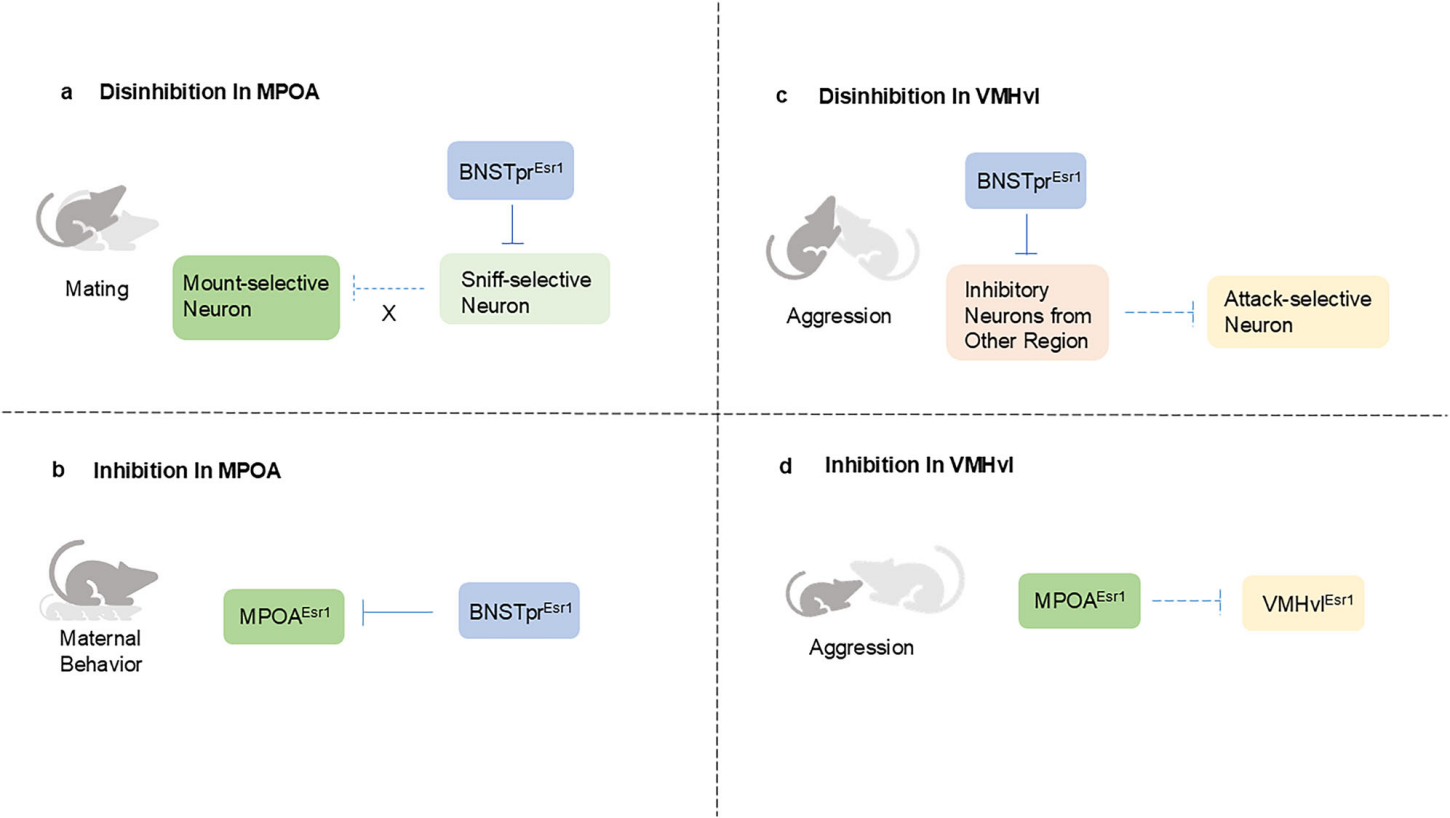
Disinhibition and inhibition in MPOA and VMHvl. In the mating context, BNSTpr^Esr1^ inhibits sniff-selective neurons in male mice MPOA, unblocking the inhibition of mount-selective neurons (***a***). BNSTpr^Esr1^ functions as a brake on maternal behavior by inhibiting MPOA^Esr1^ in female mice (***b***). In the context of the attack, BNSTpr^Esr1^ relieves the inhibitory effect of other areas on attack-selective neurons in VMHvl in male mice (***c***). To reduce aggression in male mice, MPOA^Esr1^ from the caudal part of MPOA arrests VMHvl^Esr1^ (***d***).

Mating and aggression in male mice can be suppressed by optogenetically and chemogenetially silencing BNSTpr^Esr1^ (B. Yang et al., 2022). In addition to this, inhibition of BNSTpr^Tac1^, a subset of BNSTpr^Esr1^ but smaller than BNSTpr^Aro^ (Knoedler et al., 2022; Bayless et al., 2023), greatly reduces mating and aggression of male mice. It works on POA^Tacr1^ to foster mating through the release of substance P. Furthermore, BNSTpr^Esr1^ neurons also contribute to aggressive behavior in female mice. Mei et al. employed optogenetic activation of BNSTpr^Esr1^ in noninfanticidal female mice, which projected onto MPOA and markedly triggered infanticide and suppressed maternal behavior during lactation. Maternal behavior of female mice returned to normal once light stimulation ceased (Mei et al., 2023; Fig. 2*b*). Infanticide can be considered a manifestation of aggressive behavior in female mice (Zhu et al., 2021). Thus, BNSTpr plays a gating role in mating and aggression (Fig. 1). However, the impact of BNSTpr on the hypothalamus in relation to these behaviors remains unknown.

Given that Joseff et al. identified a derepression mode of gene regulation in Esr1 neurons (Knoedler et al., 2022), and that Jiang and Pan summarized the necessary derepression in several behavior selection models in male mice and flies (Jiang and Pan, 2022), it is reasonable to assume that the repressive inputs from other regions modulate the activity of the behavior-selective cells in these two nuclei. In order to better understand how mating and aggression move from the appetitive stage to the consummatory stage, researchers identified the neurons active during appetitive behavior (sniffing) as appetitive (sniff)-selective neurons and the neurons active during consummatory behavior (mating and aggression) as consummatory behavior (mount and attack)-selective neurons (B. Yang et al., 2022). Additionally, sniff- and mount-selective neurons are separated in MPOA, and sniff- and attack-selective neurons are mixed in VMHvl. During mating or aggression of male mice, the activity of mount-selective neurons or the attack-selective neurons was significantly higher than that of sniff-selective neurons. However, the situation was reversed during sniffing. And silencing of BNSTpr^Esr1^ neurons in male mice leads to a decrease in activity of mount-selective neurons in MPOA and sniff- and attack-selective neurons in VMHvl.

The dynamic and reversible shift in male mice's behavior from appetitive to consummatory behavior is intimately associated with the activity of certain selective neurons. To control the activity of these neurons, various nuclei from the social behavior neural network transfer neurotransmitters with various characteristics. For this reason, it appears that the restriction from sniff-selective neurons to mount-selective neurons is blocked by inhibitory input from BNSTpr (Fig. 2*a*). Similarly, inhibitory inputs from other regions simultaneously suppress sniff-selective neurons and attack-selective neurons in VMHvl, which have overlapping distribution (B. Yang et al., 2022). It is possible that inhibitory input from BNSTpr counteracts this suppression (Fig. 2*c*). This was greatly supported by the discovery that optogenetic activation of GABAergic BNSTpr^Tac1^ neurons in male mice initially induced inhibition and followed by Tacr1-dependent excitatory long-term potentiation in MPOA (Bayless et al., 2023).

The majority of cell types in BNSTpr and MPOA are GABAergic, while in VMHvl they are predominantly glutamatergic (Nair et al., 2023). Cells in MPOA and VMHvl can respond to excitatory or inhibitory inputs from other regions (Mei et al., 2023). In addition to the inhibitory input of BNSTpr^Esr1^ mentioned previously, there is also a reciprocal inhibitory projection between MPOA and VMHvl at the hypothalamic level. Activation of this inhibitory projection leads to corresponding behavior effects. Specifically, activation of projection from MPOA to VMHvl decreases aggression, while activation of VMHvl to MPOA projection leads to female-directed ultrasonic vocalization emission and suppression of mating in male mice. Recent breakthroughs have shed light on the effects and significance of these projections. Some studies have shown that VMHvl receives inhibitory inputs from other regions, including MPOA in male mice (Wei et al., 2023; Minakuchi et al., 2024). Wei et al. found that by constraining VMHvl^Esr1^, cMPOA^Esr1^ (the caudal part of MPOA) can suppress male aggression (Wei et al., 2023; Fig. 2*d*). Patch clamp in conjunction with optogenetic activation, which recorded the inhibitory input from cMPOA^Esr1^ to VMHvl^Esr1^, provides evidence for this. Similar to this, Minakuchi et al. displayed that all VMHvl core neurons receive functional long-range GABAergic input from MPOA after optogenetic activation of GABAergic neurons in MPOA, resulting in optical inhibitory postsynaptic current (Minakuchi et al., 2024).

Although the sources of GABAergic input are different, all of these inhibitory inputs have varying effects on mating and aggression and are designed to facilitate defense and reproduction in mice. At the hypothalamic level, MPOA and VMHvl control the mating and aggression of male mice in distinct ways. The neurons in VMHvl are predominantly glutaminergic, and the connections between excitatory neurons generally exhibit sustained activity. In contrast, neurons in MPOA are mostly GABAergic neurons, and a subset of inhibitory neurons cannot achieve similar sustained activity (Nair et al., 2023). This distinction is closely related to the interplay between behavior selection and defense mechanisms. The all-or-nothing nature of ejaculation serves as a stage in mating to guarantee that reproduction is carried out under the proper conditions. While an attack is a periodic event, the intensity of the attack is different, in order to “bully the soft and fear the hard.” However, interestingly, during mating and aggression of male mice, certain subsets of Esr1-expressing neurons in both clusters show comparable activation (Nair et al., 2023). This nonsex-specific neuron activity suggests that their function extends beyond behavior regulation and may be involved in motivation that results in behavior to keep the neural system in a stable and continuous state conducive to defense and reproduction.

## Discussion

Overall, the analysis of Esr1-expressing neurons in the social behavior network has gradually revealed the transmission of sex cues (Fig. 1), and these findings indicate the importance of Esr1-expressing neurons in the BNSTpr and hypothalamus in encoding mating and aggression. Even though they perform differently, the modifications in neural representation specific to sex and behavior within each nucleus are closely linked to promoting defense and reproduction. Further research is necessary to refine the map of neuronal circuits that control aggression and mating at the cellular level. BNSTpr, MPOA, and VMHvl exhibit significant divergence and convergence in the process of mating and aggression, and the antagonistic or promoting properties among them require refinement. The current study focuses on the subsets of BNSTpr^Esr1^ neurons that control social behavior, providing new insights into the molecular mechanisms (Bayless et al., 2023).

Nevertheless, the majority of earlier research were obtained using mice and a single social object. In actuality, mice live in intricate social environments in their typical habitats. Improving social complexity and even simulating the natural environment are crucial for the validity and richness of the experimental results, since they bring the outcomes closer to the natural state. To go one step further, we can even release mice with implanted devices into the wild to track the brain activity of genuinely wild male mice during mating and aggression. It would be more complicated but also valuable.

However, the explanation above is restricted to mouse models. In fact, several studies have been conducted on the neural circuit associated with aggression and mating in various species. It is surprising that pC1 (posterior cell 1) neurons and VMHvl^Esr1^ neurons regulate the sexual behavior and aggression of male mice in comparable ways (Anderson, 2016). Sniffing, mating, and aggression in male mice can be gradually elicited by progressively increasing the intensity of optogenetic stimulation of VMHvl^Esr1^, and this regulating impact is dependent on neuronal population activity of VMHvl^Esr1^ (Lee et al., 2014; Nair et al., 2023). Interestingly, mating and aggression in flies are also regulated by the activity level of pC1 neurons, but the threshold dependency of aggression and mating in flies and mice is reversed (Anderson, 2016; Jiang and Pan, 2022). Additionally, VMHvl^Esr1^ neurons express tachykinin 1 that is concerned with aggression in several mammals, including humans (Shaikh et al., 1993; De Felipe et al., 1998; Coccaro et al., 2012). It is also expressed in flies and promotes aggressive motivational states of them. This shows that there may be some conservation in the neuromodulation of aggression (Lee et al., 2014; Anderson, 2016).

As more conserved molecular mechanisms and functions of neuronal activity are discovered, it is possible for us to find new targets or strategies to treat or ameliorate psychiatric disorders associated with pathological mating and aggression. In other words, research on the neural circuits governing mating and aggression can improve our comprehension of the fundamental mechanisms underlying innate behavior network and possibly provide new therapeutic avenues for the regulation of social function.

## Synthesis

Reviewing Editor: Mark Laubach, American University Decisions are customarily a result of the Reviewing Editor and the peer reviewers coming together and discussing their recommendations until a consensus is reached. When revisions are invited, a fact-based synthesis statement explaining their decision and outlining what is needed to prepare a revision will be listed below. The following reviewer(s) agreed to reveal their identity: NONE.

Two experts reviewed your manuscript and suggested the revisions outlined below. Please revise accordingly. Thank you for sending your work to eNeuro!

Reviewer #1

This review provides a succinct summary of recent cutting-edge research on the circuitry involved in mouse aggression and mating, with a focus on the role of neurons positive for estrogen receptor alpha (Esr1) located in nodes of the well-established social behavior network. The behavioral phenomena that the current results help explain are that aggression has to be inhibited in order for males to mate successfully, yet access to females may require succeeding in aggressive competition with other males. The circuitry that is summarized in Figure 1 helps explain how the switching between the two kinds of behavior (the reciprocal inhibition between aggression and mating) is possible even though both involve Esr1 neurons. The details of this circuitry are of interest both as the neural basis of an important behavioral phenomenon, and as a reminder that estrogenic mechanisms can be important for male behavior, a fact that is still not sufficiently widely known and appreciated. The review serves a useful purpose and is clear and informative for the most part, including the figure.

I do think a few minor revisions would further increase the value of the review, as follows.

The review clearly intends to focus solely on recent work at the cellular level. Nonetheless, the absence of any reference to a broader context for the work seems unfortunate. That context consists of decades of work on the role of estrogens, estrogen receptors, and of the brain nuclei of the social behavior network in the behavior of male mammals, not just mice, but a few other species as well. This becomes particularly relevant for an issue that the discussion section of the review does not address but should. The issue is whether any of the mouse work will generalize beyond that one species. The discussion section of the review includes a formulaic statement about how this work will lead to treatments for the pathological aggressive behavior of people with autism spectrum disorder or schizophrenia. This is science fiction, not science, unless there is the possibility of generality beyond this one species. Presumably generality is more likely in species where estrogen-related mechanisms have been shown to be involved in male social behavior such as mating or aggression. What are those others? Are there any primates? Are estrogen receptors thought to be involved in any human male behavior? Some reference to a comprehensive and taxonomically broader review is needed. I appreciate that for technical reasons, much of the cellular-level work has been done in mice, but that inevitably comes with some serious limitations for generality that should not be ignored.

The discussion also emphasizes the need for future research and further refinements. This is overly vague. What kinds of refinements and what new directions should be undertaken? What about the possibility of doing this kind of work with more complex and less artificial social situations? If all the work to date has tested males with a single other individual, male or female, would the same results be obtained in a test with multiple males and females such as in the social environment of mice out in the world?

Lines 132-142 are potentially interesting but some clarification would be helpful. In 136-137, do you mean that ejaculation is an all-or-none event or reflex, whereas aggression occurs is a graded behavior? I found lines 141-142 difficult to understand. Part of the difficulty is that here and in several other places in the review, "survival" is used without specifying what kind of survival. I think the authors usually mean social survival, that is, succeeding in (surviving) an aggressive encounter, but I wasn't sure here. "Survival" also includes functions that are not the subject of this review such as finding food and dealing with extreme temperatures.

Lines 14 and 162 state that the function of the circuitry is species perpetuation. That is an obsolete notion, however, based on an incorrect assumption of an overly strong group selection process. Mice are not a eusocial species, and the predominant function is the survival and reproductive success of the individual, not the species.

Reviewer #2

This manuscript discusses the role of estrogen receptor alpha (ESR1) expressing neurons within the bed nucleus of the stria terminalis (BNSTpr), medial preoptic area (MPOA) and regions of the ventromedial hypothalamus (VMH) on gating aggression and mating behavior. The suggestion that ESR1 expressing neurons within these areas are essential for mating and aggression. While this is known, they provide a model in which the medial amygdala (MeA) sends olfactory information to the BSTpr, which then sends signals to the BNSTpr. The BNSTpr then provides information to the MPOA and the VMHvl. While there is overlap in function between these areas, they suggest a model in which the MPOA regulates mating behavior and the VMHvl regulates aggression. This model is mainly discussed in terms of connections and activation or inhibition of these pathways on behavior. While the suggested model is intriguing, there needs to be more extensive and detailed discussion of the literature to support the model. Specifically, suggested paradigms should be clarified and cited. For example, the term sniff neuron is used, but no details are given as to why they are referred to as such. See below.

1) While the manuscript discusses behavioral data in regard to aggression and mating, it is not clear what sex is represented or being discussed. For example, the VMHvl is an extremely important region for regulating female rat sexual behavior, but it is discussed in the context of male sexual behavior.

2) While the title and intent of the paper is to discuss the role of ESR1 in modulating pathways between the BNSTpr, MPOA, and VMH, much of the discussion is related to glutamate and gabaergic pathways in the context of excitation and inhibition of brain regions.

3) The behaviors are not actually defined in most of the discussion. For example, mating behavior is categorized as appetitive and consummatory, such as proceptive and receptive behavior. They use sweeping terms like mating behavior, but it is clear that the cellular circuitry is different between those behaviors and how they fit into the model.

4) It is also not clear what are male preferring and female preferring neurons. Please inform the reader as to how these terms are applied and in what type of context. It is assumed perhaps in a mating context, such as a mating choice paradigm, but this is not clear.

5) The article is mostly about mating and aggression, and the authors suggest that these data may lead to the discovery of novel targets and treatments for autism spectrum disorders and schizophrenia. This may be an overstatement without further discussion of how mating behavior and aggression circuitry related to variations in social interactions associated with autism and schizophrenia. Also, in the last sentence, what is implied by the wording of new therapeutic avenues for enhanced social function in the context of mating and aggression?

6) The figure should be redesigned. It is not clear how best to accomplish this. Maybe have two figures? As the figure seems complicated in some themes and over simplified in others. One figure may contain specific brain regions and pathways, while another figure may contain behaviors and associated inhibitions and disinhibitions.

## Author Response

Response to Reviewers' Comments Manuscript Number: eN-OPN-0218-24 Paper Title: Estrogen receptor alpha expressing neurons in bed nucleus of stria terminalis and hypothalamus encoding aggression and mating General response: We sincerely thank the editors and all reviewers for their valuable time and effort to make such insightful suggestions for this manuscript, which are essential for us to improve the quality of the manuscript. The reviewer's comments are listed below in italics, with specific questions numbered. Our answers are in blue font, with changes to the original highlighted in yellow.

One-to-one response to reviewers' comments:

Reviewer #1 Comment 1: This review provides a succinct summary of recent cutting-edge research on the circuitry involved in mouse aggression and mating, with a focus on the role of neurons positive for estrogen receptor alpha (Esr1) located in nodes of the well-established social behavior network. The behavioral phenomena that the current results help explain are that aggression has to be inhibited in order for males to mate successfully, yet access to females may require succeeding in aggressive competition with other males. The circuitry that is summarized in Figure 1 helps explain how the switching between the two kinds of behavior (the reciprocal inhibition between aggression and mating) is possible even though both involve Esr1 neurons. The details of this circuitry are of interest both as the neural basis of an important behavioral phenomenon, and as a reminder that estrogenic mechanisms can be important for male behavior, a fact that is still not sufficiently widely known and appreciated. The review serves a useful purpose and is clear and informative for the most part, including the figure.

Response1: We thank the reviewers for their very positive and constructive comments, which greatly helped us to improve the quality of our manuscript. At your suggestion, we have added a wider background and an explanation of some details that are necessary for readers to understand the content of the manuscript, and at the same time, our discussion has become more rigorous. Thank you for taking time out of your busy schedule to give us such valuable advice.

Comment 2: The review clearly intends to focus solely on recent work at the cellular level. Nonetheless, the absence of any reference to a broader context for the work seems unfortunate. That context consists of decades of work on the role of estrogens, estrogen receptors, and of the brain nuclei of the social behavior network in the behavior of male mammals, not just mice, but a few other species as well. This becomes particularly relevant for an issue that the discussion section of the review does not address but should. The issue is whether any of the mouse work will generalize beyond that one species. The discussion section of the review includes a formulaic statement about how this work will lead to treatments for the pathological aggressive behavior of people with autism spectrum disorder or schizophrenia. This is science fiction, not science, unless there is the possibility of generality beyond this one species. Presumably generality is more likely in species where estrogen-related mechanisms have been shown to be involved in male social behavior such as mating or aggression. What are those others? Are there any primates? Are estrogen receptors thought to be involved in any human male behavior? Some reference to a comprehensive and taxonomically broader review is needed. I appreciate that for technical reasons, much of the cellular-level work has been done in mice, but that inevitably comes with some serious limitations for generality that should not be ignored.

Response 2: Thank you very much for your valuable input: supplementing decades of work on the role of estrogens, estrogen receptors, and of the brain nuclei of the social behavior network in the behavior of male mammals, not just mice, but a few other species as well. A good background helps the reader to understand the main content of the article. Due to the limitation of the type of paper, our summary of the background may be relatively concise, but we have made the necessary summary from these four aspects as far as possible. Since we are primarily concerned with neural networks that regulate mating and aggression in mice, we summarize only the role of estrogen, estrogen receptors, and social behavioral networks in male behavior in mice (see lines 33-65, para 1, 2, 3). Besides, only when mating and aggression neural network formed by Esr1 neurons are sufficiently conserved across species and are also fairly conserved in humans, it means that this will have an impact on aggression and mating behavior in human males. Therefore, we add to this discussion the similarities in mating and aggression between other species and mice (see lines 198 to 215, para 13). Thank you for your comments, so that our manuscript structure is more reasonable and the content is more rigorous.

Comment 3: The discussion also emphasizes the need for future research and further refinements. This is overly vague. What kinds of refinements and what new directions should be undertaken? What about the possibility of doing this kind of work with more complex and less artificial social situations? If all the work to date has tested males with a single other individual, male or female, would the same results be obtained in a test with multiple males and females such as in the social environment of mice out in the world? Response 3: We acknowledge that we were too succinct in our discussion of the need for future research and further refinements. This advice is of great value to us. Under your suggestions, we will discuss the direction and measures of future research at two aspects. Firstly, we debate the importance of summarizing conservative mechanisms of mating and aggression between different species, an area that will require significant attention in the future (see lines 198 to 213, para. 13). Secondly, it is essential to improve the social complexity and even simulate the natural environment to obtain the experimental results closer to the natural state. Going one step further, we can even release mice with implantable devices into the wild to monitor neural activity in mating and aggression states in truly natural male mice (see lines 190 to 197, para. 12). Transformation of neural representation at this time may involve a mix of multiple behavioral selection, as well as an advance or delay in aggression and mating caused by selection weights in different environments. This will be a composite result of multiple behavioral choices. Thank you very much for your attention.

Comment 4: Lines 132-142 are potentially interesting but some clarification would be helpful. In 136-137, do you mean that ejaculation is an all-or-none event or reflex, whereas aggression occurs is a graded behavior? I found lines 141-142 difficult to understand. Part of the difficulty is that here and in several other places in the review, "survival" is used without specifying what kind of survival. I think the authors usually mean social survival, that is, succeeding in (surviving) an aggressive encounter, but I wasn't sure here. "Survival" also includes functions that are not the subject of this review such as finding food and dealing with extreme temperatures.

Response 4: Thank you very much for your suggestion, and we apologize for not being clear enough to cause you any doubt here. First of all, we are did trying to say that "ejaculation is an all-or-none event or reflex, whereas aggression occurs is a graded behavior". We have changed to "The all-or-nothing nature of ejaculation serves as a stage in mating to guarantee that reproduction is carried out under the proper conditions. While attack is a periodic event, the intensity of the attack is different, in order to "bully the soft and fear the hard" (see lines 168 to 171, para. 10). Secondly, the word "survival" mentioned in lines 141-142 of the original manuscript is intended to convey three meanings. First, the mice actively defend their territory by attacking intruders of the same sex. Second, male mice faced with a strong competitor does not actively attack to avoid serious injury. Third, male mice engage in mating behavior with female mice, thus achieving the reproduction. The first two are both defensive behaviors, which are manifested in the neural circuit as aggression activation and inhibition. Thus, we have changed "animal survival" and the like to "defense and reproduction" (see line 14, 31, 162, 168, 176, 183). Thank you for your advice, which makes our manuscript more organized and smoother.

Comment 5: Lines 14 and 162 state that the function of the circuitry is species perpetuation. That is an obsolete notion, however, based on an incorrect assumption of an overly strong group selection process. Mice are not a eusocial species, and the predominant function is the survival and reproductive success of the individual, not the species.

Response 5:

We sincerely thank reviewer for valuable feedback that we have used to improve the quality of our manuscript. We have adopted this suggestion to modify the concept of species perpetuation to defense and reproduction (see line 14, 31, 162, 168, 176, 183). Our intention is that the neural network of mating and aggression in male mice is designed to encourage individuals to behave in accordance with external chemical signals that are appropriate for defense and reproduction of individual male mice. In the original article, we extended the concept of individuals to species, which causes unnecessary misunderstanding. We have amended "species perpetuation" to "defense and reproduction", thank you for your patient correction.

Reviewer #2 Comment 1: This manuscript discusses the role of estrogen receptor alpha (ESR1) expressing neurons within the bed nucleus of the stria terminalis (BNSTpr), medial preoptic area (MPOA) and regions of the ventromedial hypothalamus (VMH) on gating aggression and mating behavior. The suggestion that ESR1 expressing neurons within these areas are essential for mating and aggression. While this is known, they provide a model in which the medial amygdala (MeA) sends olfactory information to the BSTpr, which then sends signals to the BNSTpr. The BNSTpr then provides information to the MPOA and the VMHvl. While there is overlap in function between these areas, they suggest a model in which the MPOA regulates mating behavior and the VMHvl regulates aggression. This model is mainly discussed in terms of connections and activation or inhibition of these pathways on behavior. While the suggested model is intriguing, there needs to be more extensive and detailed discussion of the literature to support the model. Specifically, suggested paradigms should be clarified and cited. For example, the term sniff neuron is used, but no details are given as to why they are referred to as such. See below.

Response 1: Thank you for your recognition of the model used in this manuscript, and we greatly appreciate your suggestions and valuable time on the manuscript. We have added a broader discussion including the role of estrogens, estrogen receptors, and of the brain nuclei of the social behavior network to complete the background (see lines 33 to 65, para. 1, 2, 3). Besides, we have also clarified the context of some terms used in the text. For example, we added the background of sniff-selective neurons, mount selective neurons, attack selective neurons, appetitive behavior selective neurons, and consummatory behavior selective neurons in our manuscript (see lines 118 to 122, para. 7).

Comment 2: While the manuscript discusses behavioral data in regard to aggression and mating, it is not clear what sex is represented or being discussed. For example, the VMHvl is an extremely important region for regulating female rat sexual behavior, but it is discussed in the context of male sexual behavior.

Response 2: Thank you for your insightful suggestions here. Firstly, what we are mainly talking about in this manuscript is mating and aggression in male mice. Besides, in order to enrich the mechanisms of neuronal inhibition and disinhibition, the infanticide and maternal behavior we mentioned are discussed in female mice. To avoid ambiguity, we have added sex and species where appropriate to avoid confusion for readers. (see line 3, 76, 85, 88, 98, 107, 108, 109, 115, 124, 129, 149, 152, 163, 172).

Comment 3: While the title and intent of the paper is to discuss the role of ESR1 in modulating pathways between the BNSTpr, MPOA, and VMH, much of the discussion is related to glutamate and gabaergic pathways in the context of excitation and inhibition of brain regions.

Response 3: Thank you very much for such precious comments, which are very valuable to the structure of our article. We want to show that nuclei from the social behavior neural network regulate the activity of different selective neurons by neurotransmitters. In fact, in the process of sorting out and summarizing the references, we found that Esr1 neurons in mating and aggression neural network not only have regulatory effects on other neurons, but they are also regulated by Esr1 neurons in other nuclei. At the same time, Esr1 neurons are GABAergic or glutaminergic neurons. It is the regulation between GABAergic and glutamergic Esr1 neurons in BNSTpr, VMHvl, MPOA that enables the control of mating and aggression. Here, we have added the transition section (see lines 129 to 132, para. 8) to make the transition between "the role of ESR1 in modulating pathways between the BNSTpr, MPOA, and VMH" and "glutamate and gabaergic pathways in the context of excitation and inhibition of brain regions" more natural and smoother. We have not expressed this clearly before, and we appreciate your valuable input.

Comment 4: The behaviors are not actually defined in most of the discussion. For example, mating behavior is categorized as appetitive and consummatory, such as proceptive and receptive behavior. They use sweeping terms like mating behavior, but it is clear that the cellular circuitry is different between those behaviors and how they fit into the model.

Response 4: Thank you for your kind advices to us. We have added to the beginning of the manuscript the explanation that sniffing belongs to the appetitive behavior, mating and aggression belong to consummatory behavior of male mice, in order to define the mating and aggression mentioned in the whole article (see lines 33 to 34, para. 1). Thank you for your comments.

Comment 5: It is also not clear what are male preferring and female preferring neurons. Please inform the reader as to how these terms are applied and in what type of context. It is assumed perhaps in a mating context, such as a mating choice paradigm, but this is not clear.

Response 5: We sincerely thank you for your suggestions on the context of the use of the term. We have accepted your comment and added the background and origin of the female-preferring neurons and male-preferring neurons to the new manuscript (see lines 79 to 84, para. 5). In the study of the sex-specific neural representation of BNSTpr in male mice, the researchers suspended mice of different sexes in male mouse cages. During this time, male mice are only allowed to sniff, not mount or attack. They defined female preferring neurons and male preferring neurons as those that were active only when sniffing female or male mice, respectively. Thus, female preferring neurons and male preferring neurons are defined in the context of sniffing of male mice, not aggression or mating. Before this, we omit the introduction when quoting terms from other literature, which is easy to mislead readers. Hence, we appreciate your attention to this important detail.

Comment 6: The article is mostly about mating and aggression, and the authors suggest that these data may lead to the discovery of novel targets and treatments for autism spectrum disorders and schizophrenia. This may be an overstatement without further discussion of how mating behavior and aggression circuitry related to variations in social interactions associated with autism and schizophrenia. Also, in the last sentence, what is implied by the wording of new therapeutic avenues for enhanced social function in the context of mating and aggression? Response 6: We thank the reviewer for pointing this out. In the discussion section of the manuscript, we have added the similarities and differences between males of different species in dealing with aggression and mating, and added a literature related to aggression in human personality disorders, which complements the possibility of extending the research on mating and aggression to the treatment of human pathological mating and aggression (see lines 198 to 215 para, 13, 14 and reference. 39). Until then, we must admit that it would be too hasty to extend the results of the research on mice to the possibility of treating human diseases without discussing the similarities and conservative mechanisms of mating and aggression between species. Only if the mating and aggression neural network formed by Esr1 neurons is sufficiently conserved across species, and is also quite conserved in humans, does it mean that it has an impact on mating and aggression in human males. Therefore, in the context of mating and aggression, building on the current results of such a broad range of studies, the future will be able to discover more in-depth cellular and molecular conservative mechanisms of Esr1-expressing neurons. When this conservative mechanism is extended to humans, intervention in Esr1-expressing neurons will affect mating and aggression of male humans.

Comment 7: The figure should be redesigned. It is not clear how best to accomplish this. Maybe have two figures? As the figure seems complicated in some themes and over simplified in others. One figure may contain specific brain regions and pathways, while another figure may contain behaviors and associated inhibitions and disinhibitions.

Response 7: According to your suggestion, we have modified Fig 1 in the original article and split it into two diagrams. The new Fig. 1 shows how sex cues travel through BNSTpr, MPOA and VMHvl involved in controlling mating and aggression (see new Fig. 1 and line 316 to 322). The other shows the inhibition and disinhibition mechanisms that control behavior choice (see new Fig. 2 and line 324 to 329). We have placed the revised two images in the article to ensure that they effectively convey the intended information to the reader. Thank you very much for your attention to this detail.
